# Shift work, sociodemographic factors, and sleep quality among nurses working in rotating and day shifts: a cross-sectional analysis

**DOI:** 10.3389/fpsyt.2026.1860489

**Published:** 2026-09-03

**Authors:** Maaidah M. Algamdi, Mashael Hasan Alamrani

**Affiliations:** 1 Community and Psychiatric Health Nursing Department, Faculty of Nursing, University of Tabuk, Tabuk, Saudi Arabia; 2 Nursing Administration and Education Department, Faculty of Nursing, University of Tabuk, Tabuk, Saudi Arabia

**Keywords:** cross-sectional association, logistic regression, nurses, PSQI, rotating shifts, Saudi Arabia, shift work, sleep quality

## Abstract

**Background:**

Nurses’ sleep quality is vital for their health and well-being, especially given their demanding, irregular schedules. This study examined associations between work schedule, shift selection, and sociodemographic factors and sleep quality among nurses in Saudi Arabia.

**Methods:**

A self-reported, descriptive cross-sectional study used a two-part questionnaire comprising sociodemographic data and the Pittsburgh Sleep Quality Index (internal consistency α = .824). Chi-square tests and Cramér’s V assessed bivariate associations between demographic/workplace variables and sleep quality. Because several candidate predictors (education, experience, age) were severely collinear with the shift-exposure variables (variance inflation factors up to 14) and several subgroups showed near-deterministic relationships with the outcome, a parsimonious multivariable logistic regression adjusted for nationality and marital status, estimated using Firth’s penalized (bias-reduced) method, replaced the originally planned partial least squares structural equation modeling mediation analysis, which assumed a temporally implausible causal ordering for cross-sectional data.

**Results:**

Of 140 nurses analyzed, 60 (42.9%) reported good sleep quality and 80 (57.1%) reported poor sleep quality. Good sleep quality declined with age (70.0% at 20–24 years to 24.5% at >35 years; χ² (3) = 11.85, p = .008). Work schedule, shift selection, education, experience, department, marital status, and nationality were all significantly associated with sleep quality (all p <.001; Cramér’s V 0.42–0.78). After adjustment, nurses on rotating shifts had substantially lower odds of poor sleep quality than those on fixed daytime schedules (OR = 0.02, 95% CI [0.00, 0.10], p <.001), and nurses mandatorily assigned to their shift had lower odds of poor sleep than those who selected their shift voluntarily (OR = 0.14, 95% CI [0.03, 0.61], p = .009).

**Conclusion:**

Work schedule and shift selection were the strongest correlates of sleep quality in this sample, but these associations, including the direction of the rotating-shift and mandatory-assignment findings, are cross-sectional and adjusted, not causal, and should be interpreted alongside the severe collinearity among education, experience, age, and shift assignment. Prospective research is needed to clarify these relationships before informing scheduling policy.

## 1 Introduction

Nurses, as healthcare professionals, play a crucial role in delivering continuous care and support within the healthcare system. Their work requires collaboration with multidisciplinary teams to promote health and provide nursing care to individuals, families, and communities. However, the demanding nature of their profession—characterized by irregular schedules, extended shifts, and night work—significantly disrupts circadian rhythms and affects sleep quality (1). Circadian rhythms are internal biological processes that regulate the sleep-wake cycle, repeating approximately every 24 hours (2). The disruption of these rhythms due to night shifts and rotating schedules has been linked to poor sleep quality, negatively impacting nurses’ overall health and quality of life (3). Understanding the effects of shift-related sleep disturbances is essential for developing interventions that can enhance well-being and optimize patient care outcomes (4).

Sleep quality is a key determinant of physical and mental health, particularly for healthcare professionals such as nurses who frequently encounter unpredictable and demanding work schedules (5). The nursing profession involves long working hours, frequent night duties, and minimal recovery time between shifts, all of which pose significant challenges to maintaining adequate sleep (3, 5). Chang et al. (6) underscore the need to identify and address factors influencing nurses’ sleep patterns, as poor sleep quality has been associated with increased medical errors, burnout, and adverse health outcomes. Sleep deprivation in nurses can lead to fatigue, impaired decision-making, and reduced alertness, which may compromise both personal well-being and patient safety (7, 8). Nurses working irregular shifts with minimal recovery time—especially those alternating between morning, afternoon, and night shifts within the same week—are at greater risk of insomnia and sleep disturbances (9).

Adequate sleep is essential for cognitive function, emotional stability, and physical health, directly influencing job performance and patient safety (10, 11). Consequently, reliable sleep assessment tools are necessary for research. The PSQI is a widely used instrument that evaluates sleep duration, disturbances, latency, and daytime dysfunction, making it a valuable measure for assessing nurses’ sleep quality (12–15). Previous studies have demonstrated that prolonged work hours elevate the risk of mental health disorders, sleep disturbances, and cardiovascular diseases among nurses (16–18).

Beyond its effects on patient safety (19), poor sleep quality poses serious health risks for nurses, increasing their susceptibility to chronic conditions such as obesity, cardiovascular disease, and mental illness (16–18). Thus, improving nurses’ sleep health is critical to promoting well-being, enhancing job satisfaction, and ensuring long-term career sustainability. Several demographic factors—including age, gender, marital status, educational level, and nationality—have been found to influence sleep quality among nurses (20). Research highlights that variations in these factors contribute to different sleep patterns, necessitating tailored interventions to address sleep disparities among nursing professionals (21, 22). Additionally, workplace-related factors such as shift schedules, workload, and clinical responsibilities also impact sleep quality. Evidence suggests that non-standard work hours and rotating shifts significantly impair sleep, reinforcing the importance of investigating how work environments and shift patterns affect sleep quality in nurses (23).

Much of the existing literature, and the original design of this study, has framed sociodemographic variables such as education and years of experience as mediators of the relationship between shift assignment and sleep quality. On reflection, and as raised during peer review, this framing assumes that current shift assignment can influence attainment that was, in nearly all cases, completed before that assignment began an implausible temporal sequence in cross-sectional data. Because education, experience, and age instead plausibly influence which shift a nurse is assigned to (a relationship this study’s own data directly support; this study adopts an adjusted cross-sectional association approach: education, experience, age, and nationality are treated as antecedent covariates and confounders rather than as mediating mechanisms. The aim of this study is therefore to examine the cross-sectional associations of work schedule, shift selection, and sociodemographic factors with the sleep quality of nurses in Saudi Arabia, adjusting for confounding where the data permit.

## 2 Theoretical framework

The Two-Process Model of Sleep Regulation (24, 25) motivated this study’s interest in shift work as a correlate of sleep quality. This widely applied model in sleep research proposes that sleep is governed by Process C (circadian rhythm regulation) and Process S (homeostatic sleep drive). Circadian rhythms regulate the sleep-wake cycle, promoting wakefulness during the day and sleep at night; night shifts and rotating schedules can disrupt this cycle, in principle causing misaligned sleep timing, reduced sleep duration, and increased fatigue due to insufficient recovery time. Borbély et al. (25) highlight how the continuous interaction between Processes S and C plays a crucial role in performance, fatigue, and individual sleep regulation differences.

This study does not measure circadian phase, homeostatic sleep pressure, or any other biological indicator of Process C or Process S; it measures only organizational (work schedule, shift selection) and sociodemographic correlates of self-reported sleep quality. The Two-Process Model is therefore used here strictly as background motivation for why shift work might plausibly relate to sleep specifically the Work Schedule and not as a mechanism that this study’s design or data can test directly. Associations involving education, experience, nationality, marital status, or department are sociodemographic and organizational in nature and are not interpreted through this biological framework.

## 3 Materials and methods

### 3.1 Study design and setting

A descriptive cross-sectional, self-reported study was conducted to examine how shift work relates to nurses’ sleep quality and how sociodemographic factors are associated with sleep outcomes. The study was conducted across multiple hospitals in the Tabuk region, Saudi Arabia, which adheres to Saudi Ministry of Health policies regarding shift rotation. Participating hospitals operated on a three-shift schedule: Shift A (7:00 AM–3:00 PM), Shift B (3:00 PM–11:00 PM), and Shift C (11:00 PM–7:00 AM).

### 3.2 Study sample

Eligible participants included nurses aged 20 years or older of either gender, holding at least a diploma, bachelor’s degree, or other advanced nursing qualification, and having more than 6 months of work experience. Nursing students in training or internship programs were excluded due to their regular morning shift schedules. The study targeted nurses working in both inpatient and outpatient general units.

A total of 227 nurses were initially recruited, with the sample size determined using the Raosoft Sample Size Calculator, applying a 5% margin of error, 95% confidence level, and an estimated population size of 552 nurses in the Tabuk region.

**Figure 1:**
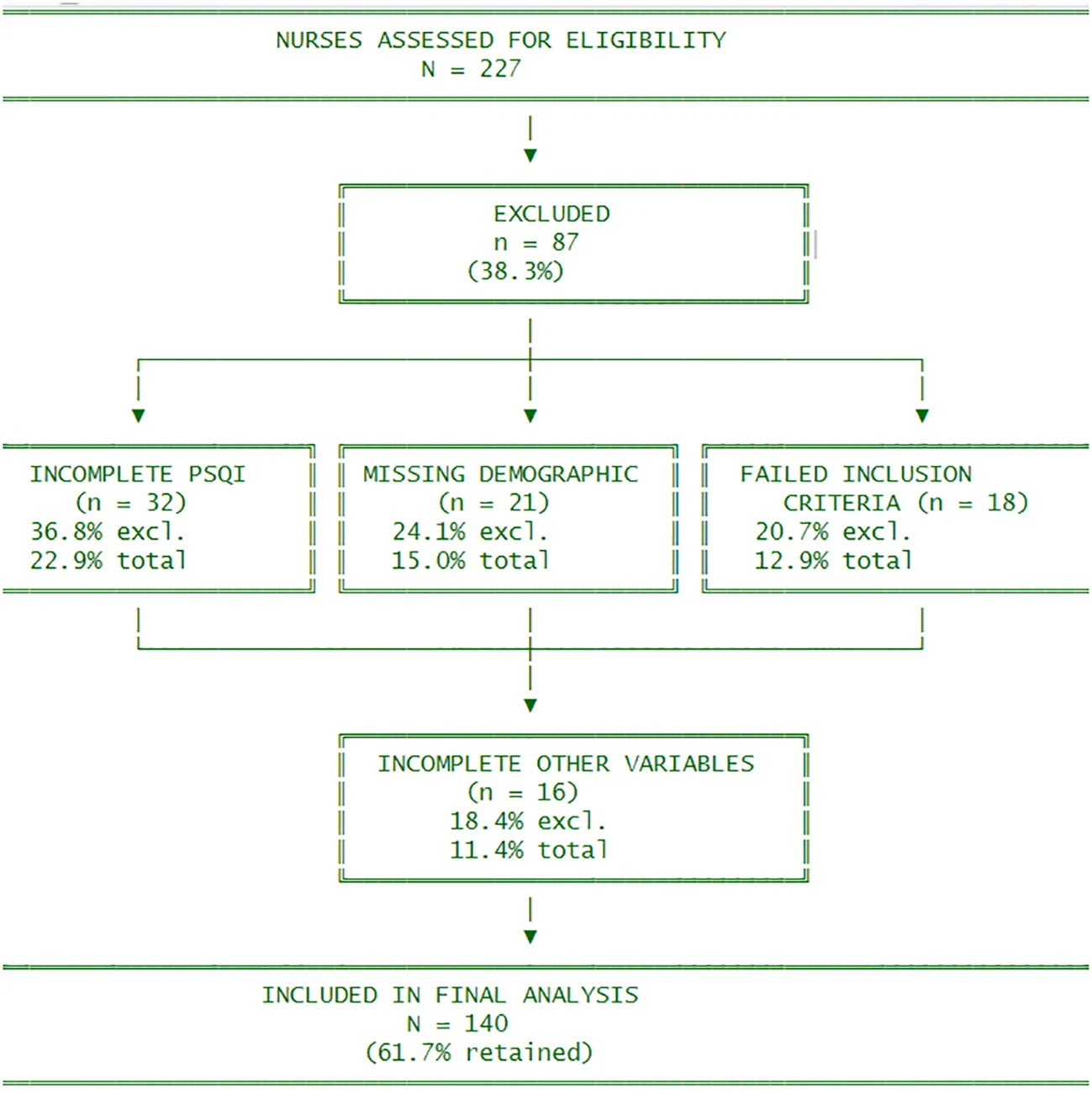
CONSORT-style flow diagram of participant recruitment and retention.

Following data collection, the returned questionnaires were screened for completeness. Of the 227 questionnaires received, 87 (38.3%) were excluded from the final analysis for the following reasons: incomplete Pittsburgh Sleep Quality Index (PSQI) responses (n = 32), missing demographic data (n = 21), failure to meet the pre-specified inclusion criteria (n = 18), and incomplete responses on other key study variables (n = 16), **Figure 1**. The final analytical sample therefore comprised 140 participants, representing a retention rate of 61.7% of the initial sample.

To assess the potential for selection bias due to participant loss, included (n = 140) and excluded (n = 87) participants were compared on available demographic characteristics. No significant differences were observed between the two groups in age, gender, nationality, department, or work schedule (all p > 0.05; data not given), suggesting that attrition did not systematically bias the sample on observable characteristics.

### 3.3 Data collection and instruments

Data were collected over a period of 4 months using a self-administered questionnaire composed of two parts. The first part gathered demographic characteristics including age, sex, marital status, department, work schedule, level of education, work experience, and common health problems. The second part employed the PSQI to assess subjective sleep quality, sleep latency, sleep duration, habitual sleep efficiency, sleep disturbances, use of sleep medication, and daytime dysfunction over the past month. The seven component scores are summed to form the PSQI global score (0–21), with higher scores indicating poorer sleep quality; a global score >5 indicates poor sleep quality (12). **Table 1** lists the standard abbreviation, full name, and content of each component score; these abbreviations are used exclusively throughout the remainder of this manuscript.

**Table 1:** PSQI component score definitions.

Abbreviation	Component	What it measures
PSQISLPQUAL	Subjective Sleep Quality	Participant’s own overall rating of their sleep (very good to very bad)
PSQIMEDS	Use of Sleeping Medication	Frequency of prescription or over-the-counter sleep aid use
PSQIDURAT	Sleep Duration	Total number of hours of sleep obtained per night
PSQIDISTB	Sleep Disturbances	Frequency of problems disrupting sleep (waking at night, bathroom use, coughing, temperature discomfort)
PSQIDAYDYS	Daytime Dysfunction	Trouble staying awake during daily activities or lack of enthusiasm to get things done
PSQILATEN	Sleep Latency	Time taken to fall asleep (≤15, 16–30, 31–60, or >60 minutes)
PSQIHSE	Habitual Sleep Efficiency	Percentage of time in bed actually spent asleep (total sleep time/time in bed × 100)

Internal consistency of the PSQI was evaluated using Cronbach’s alpha computed on the seven component scores. Reliability was good in this sample (α = .824; N = 140 valid cases, 0 excluded listwise; **Table 2**), exceeding the acceptable threshold described in prior validation work (26). This sample-specific value supersedes the general threshold statement in the original protocol.

**Table 2:** Case processing summary and reliability statistics.

Case description	N	%
Valid cases	140	100.0
Excluded (listwise)	0	0.0
Total	140	100.0
**Cronbach’s Alpha**	**N of Items**	
0.824	7	

### 3.4 Statistical analysis

Descriptive statistics (frequencies and percentages) were computed in SPSS version 27.0, as in the original protocol. Following peer review, the inferential analysis strategy was revised as follows. All demographic and workplace variables were treated as nominal or ordinal categorical variables and entered into models using explicit dummy (indicator) coding with pre-specified reference categories: Work Schedule = Daytime; Shift Selection = Voluntary; Nationality = Saudi; Marital Status = Married; Education = Bachelor; Experience = <1 year; Age = 20–24 years.

Bivariate associations between demographic/workplace variables and PSQI category (Good/Poor) were assessed using Pearson chi-square tests; Fisher’s exact test is reported as a supplement wherever any expected cell count fell below 5. Effect sizes for these nominal-by-nominal associations were quantified using Cramér’s V; Pearson product-moment correlation, used for this purpose in the original protocol, is not a valid measure of association between variables whose numeric codes carry no inherent order or spacing and was therefore replaced.

**Figure 2:**
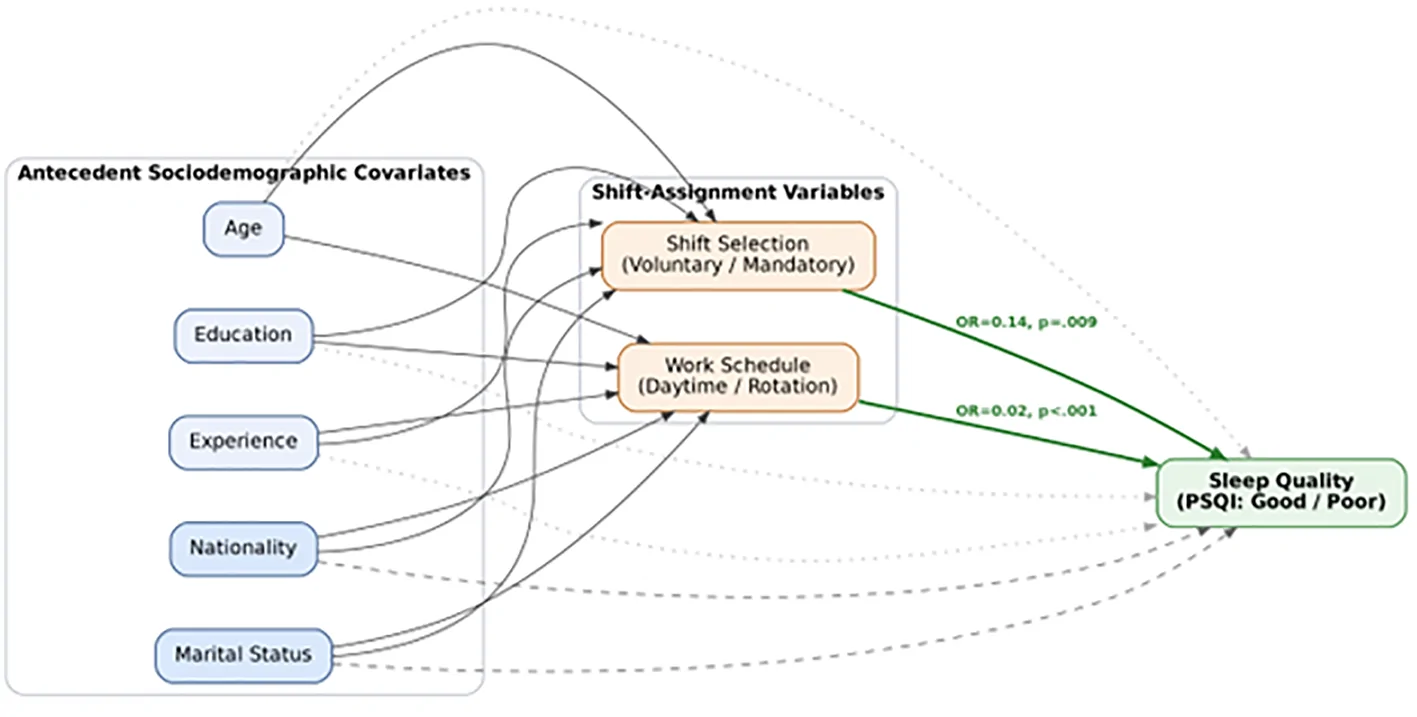
Directed acyclic graph (DAG) of the analytic model. Solid black arrows: antecedent sociodemographic covariates predicting shift assignment (**Tables 3**, **4**, all p <.001). Dotted gray arrows: Age, Education, and Experience → PSQI are bivariate/descriptive associations only (**Table 2**) these three variables could not be entered into the adjusted model jointly with the shift-exposure variables due to severe collinearity (VIF up to 14; **Table 7**). Dashed gray arrows: Nationality and Marital Status → PSQI were tested in the adjusted model and were not significant (**Table 6**). Bold green arrows: primary adjusted model estimates, both significant (**Table 6**).

Collinearity among candidate predictors was assessed using variance inflation factors (VIF) computed from the dummy-coded design matrix. Several predictors showed severe collinearity (VIF > 10) with the shift-exposure variables, and an exploratory model containing all covariates simultaneously failed to converge to stable estimates, consistent with quasi-complete separation driven by near-deterministic subgroups in the data. The originally planned mediation model (partial least squares structural equation modeling, PLS-SEM, fitted in Jamovi) assumed that current shift assignment could influence antecedent variables such as completed education and accumulated experience, a temporally implausible causal ordering; it has therefore been replaced with a parsimonious, pre-specified multivariable logistic regression for PSQI category (Poor vs. Good), adjusted for the covariates that did not show problematic collinearity (nationality, marital status), with education and experience retained as descriptive (bivariate) associations only (**Table 6**). The full analytic model, including the antecedent sociodemographic covariates, the shift-assignment variables, and their hypothesized relationships to sleep quality, is illustrated in **Figure 2**.

**Table 3:** Demographic characteristics of participants (n = 140).

Variable	Category	n	%
Age	20–24	10	7.1
	25–29	48	34.3
	30–34	33	23.6
	>35	49	35.0
Gender	Male	5	3.6
	Female	135	96.4
Marital Status	Married	91	65.0
	Single	46	32.9
	Others	3	2.1
Education	Diploma	20	14.3
	Bachelor	78	55.7
	Other	42	30.0
Experience	>6mo–<1yr	3	2.1
	1–5yr	52	37.1
	5–10yr	40	28.6
	≥10yr	45	32.1
Department	Emergency	1	0.7
	ICU	10	7.1
	Medical Ward	28	20.0
	Surgical Ward	16	11.4
	Maternity	15	10.7
	Other	70	50.0
Nationality	Saudi	80	57.1
	Non-Saudi	60	42.9
Current Job	Nurse Assistant	15	10.7
	SN2	24	17.1
	SN1	68	48.6
	CN	14	10.0
	HN	6	4.3
	Other	13	9.3
Work Schedule	Daytime	84	60.0
	Shift Rotation	56	40.0
Shift Selection	Voluntary	82	58.6
	Mandatory	58	41.4
PSQI Global Score	Good Sleep Quality	60	42.9
	Poor Sleep Quality	80	57.1

SN1, Staff Nurse Grade 1; SN2, Staff Nurse Grade 2; CN, Charge Nurse; HN, Head Nurse; PSQI, Pittsburgh Sleep Quality Index.

Given the evidence of quasi-complete separation, model parameters were estimated using Firth’s penalized (bias-reduced) logistic regression (27, 28), which yields finite, stable estimates under sparse-data and separation conditions using an asymptotic penalized-likelihood correction, without requiring bootstrap resampling. Two sensitivity models, individually adding education or experience to the primary model, were fitted to evaluate the robustness of the shift-exposure estimates. Statistical significance was set at p <.05 throughout. Chi-square, Cramér’s V, VIF, and Firth logistic regression models were fitted in R (version 4.x) using the tidyverse, vcd, logistf, and car packages; a fully reproducible analysis script is available from the corresponding author.

### 3.5 Ethical considerations

This study was conducted in accordance with the Declaration of Helsinki and received ethical approval from the General Directorate of Health Affairs Institutional Review Board (Registration No. H-07-TU-077). Permission was secured from the original authors to use the PSQI for data collection. Written informed consent was obtained from all participants, who were informed of their right to withdraw at any time. Anonymity was maintained by not collecting any identifying information. Participants meeting the inclusion criteria were invited to complete the questionnaire during their shifts, and collaboration with official bodies in each setting ensured timely distribution and collection of questionnaires.

## 4 Results

### 4.1 Demographic profile of participants

**Table 3** presents the demographic characteristics of the 140 participants. The majority were aged over 35 years (35.0%), followed by those aged 25–29 years (34.3%). Most participants were female (96.4%), and 65.0% were married. Over half held a bachelor’s degree (55.7%). The largest group had 1 to <5 years of experience (37.1%). Participants worked across various departments, with the largest proportion in other specified areas (50.0%) and medical wards (20.0%). A majority were Saudi nationals (57.1%). The most common job role was Staff Nurse Grade 1 (SN1; 48.6%). Regarding work schedule, 60.0% worked daytime shifts and 40.0% worked shift rotations; 58.6% chose their shifts voluntarily. PSQI assessment indicated that 57.1% experienced poor sleep quality (score >5) and 42.9% had good sleep quality; internal consistency of the PSQI in this sample was good (Cronbach’s α = .824; **Table 2**).

### 4.2 Demographic predictors of shift work assignment

**Table 4** presents Chi-square analyses comparing voluntary versus mandatory shift assignment across demographic variables. Age was significantly associated with shift assignment mode (χ² (3) = 19.875, p < 0.001); younger nurses (20–24 years) were more likely to receive mandatory assignments, whereas nurses aged >35 years predominantly selected shifts voluntarily. Marital status was also significantly associated with shift selection (χ² (2) = 30.994, p < 0.001), with married nurses more frequently assigned mandatory shifts and single nurses more likely to choose voluntarily. Educational attainment showed a strong significant association (χ² (2) = 86.764, p < 0.001); bachelor’s degree holders predominantly worked voluntarily, whereas nurses with other qualifications were more often mandatorily assigned. Professional experience was another significant factor (χ² (3) = 67.763, p < 0.001); nurses with ≥10 years of experience were predominantly assigned mandatory shifts, while those with <1 year of experience mainly worked voluntarily. Nationality also significantly influenced shift assignment (χ² (1) = 42.742, p < 0.001), with Saudi nurses more likely to receive mandatory assignments compared to non-Saudi nurses. These patterns directly support treating education, experience, and nationality as antecedents of shift assignment rather than as consequences of it.

### 4.3 Factors influencing daytime and rotating work schedules

**Table 5** presents the distribution of work schedule type by demographic variables. Younger nurses (20–24 years) predominantly worked rotating shifts, whereas older nurses (>35 years) mainly worked daytime shifts (χ² (3) = 22.254, p < 0.001). Married nurses were more likely to work rotating shifts, while single nurses predominantly worked daytime schedules (χ² (2) = 31.925, p < 0.001). Nurses with diplomas or bachelor’s degrees were predominantly found in daytime shifts, whereas those with other qualifications were more likely to work rotating schedules (χ² (2) = 62.277, p < 0.001). Nurses with ≥10 years of experience predominantly worked rotating shifts, while those with less than 1 year of experience mainly worked daytime schedules (χ² (3) = 75.547, p < 0.001). Saudi nurses were more likely to work rotating shifts compared to non-Saudi nurses (χ² (1) = 48.611, p < 0.001).

**Table 4:** Comparison of demographic factors by shift assignment mode (voluntary vs. mandatory).

Variable	20–24	25–29	30–34	>35	Total	χ²	df	p
Age -Voluntarily	4	21	16	41	82	19.875	3	<.001
Age -Mandatorily	6	27	17	8	58			
Marital: Married/Single/Others -Voluntarily	38	42	2	—	82	30.994	2	<.001
Marital -Mandatorily	53	4	1	—	58			
Education: Diploma/Bachelor/Other -Voluntarily	14	68	0	—	82	86.764	2	<.001
Education -Mandatorily	6	10	42	—	58			
Experience (4 groups) -Voluntarily	2	43	33	4	82	67.763	3	<.001
Experience -Mandatorily	1	9	7	41	58			
Nationality: Saudi/NonSaudi -Voluntarily	28	54	—	—	82	42.742	1	<.001
Nationality -Mandatorily	52	6	—	—	58			

Column headings correspond to each variable’s own categories (see **Table 1**); -denotes not applicable. Gender was also tested (χ² (*1*) = 0.981, p = .322, not significant) and is omitted from the table for brevity.

### 4.4 Demographic factors and their association with sleep quality

**Table 6** presents chi-square analyses of sleep quality by demographic variables. Age was significantly associated with sleep quality (χ² (3) = 11.854, p = .008, Cramér’s V = 0.29); the proportion reporting good sleep quality declined from 70.0% among nurses aged 20–24 years to 24.5% among nurses older than 35 years. This pattern reverses the interpretation given in the original submission (“sleep quality improved across older age groups”) and is instead consistent with the positive age–PSQI association reported in **Table 5** and the adjusted model in **Table 6**. Marital status was significantly associated with sleep quality (χ² (2) = 29.426, p <.001, Cramér’s V = 0.46), with married participants more likely to report good sleep quality. Educational attainment was significantly associated with sleep quality (χ² (2) = 56.125, p <.001, Cramér’s V = 0.63); participants with ‘Other’ educational qualifications reported the highest rate of good sleep quality (38/42, 90.5%). Professional experience showed a strong association (χ² (3) = 72.039, p <.001, Cramér’s V = 0.72); nurses with ≥10 years of experience had the highest rate of good sleep quality (42/45, 93.3%). Nationality was significantly associated with sleep quality (χ² (1) = 24.063, p <.001, Cramér’s V = 0.42), with Saudi nurses reporting better sleep quality than non-Saudi nurses. Because Education, Experience, and Age are strongly collinear with Work Schedule and Shift Selection (**Table 7**), the associations reported in this table are descriptive rather than adjusted or independent effects; adjusted estimates are presented in **Table 6**. Fisher’s exact test is recommended wherever expected cell counts fall below 5.

**Table 5:** Work schedule type across demographic variables.

Variable	20–24	25–29	30–34	>35	Total	χ²	df	p
Age -Daytime	3	22	17	42	84	22.254	3	<.001
Age -Rotation	7	26	16	7	56			
Marital -Daytime	39	42	3	—	84	31.925	2	<.001
Marital -Rotation	52	4	0	—	56			
Education -Daytime	12	67	5	—	84	62.277	2	<.001
Education -Rotation	8	11	37	—	56			
Experience -Daytime	1	45	34	4	84	75.547	3	<.001
Experience -Rotation	2	7	6	41	56			
Nationality -Daytime	28	56	—	—	84	48.611	1	<.001
Nationality -Rotation	52	4	—	—	56			

**Table 6:** Demographic factors and their association with sleep quality (PSQI).

Age				
Sleep Quality	20-24	25-29	30-34	>35
Good	7	25	16	12
Poor	3	23	17	37
*χ² (3*)*=11.854, p=.008, Cramér’s V = 0.29 (Fisher’s exact recommended; min. expected 4.29)*				

Marital Status				
Sleep Quality		Married	Single	Others
Good		54	5	1
Poor		37	41	2
*χ² (2*)*=29.426, p<.001, Cramér’s V = 0.46 (Fisher’s exact recommended; min. expected 1.29)*				

Education				
Sleep Quality		Diploma	Bachelor	Other
Good		3	19	38
Poor		17	59	4
*χ²(2)=56.125, p<.001, Cramér’s V = 0.63*				

Experience				
Sleep Quality	<1yr	1-5yr	5-10yr	≥10yr
Good	2	10	6	42
Poor	1	42	34	3
*χ²(3)=72.039, p<.001, Cramér’s V = 0.72 (Fisher’s exact recommended; min. expected 1.29)*				

Nationality				
Sleep Quality			Saudi	Non-Saudi
Good			49	11
Poor			31	49
*χ²(1)=24.063, p<.001, Cramér’s V = 0.42*				

PSQI, Pittsburgh Sleep Quality Index.

**Table 7:** Association measures between sleep quality (PSQI) and workplace demographics.

Variable	Cramér’s V	χ²	df	p
Work Schedule	0.78	85.343	1	<.001
Experience	0.72	72.039	3	<.001
Shift Selection	0.66	61.626	1	<.001
Education	0.63	56.125	2	<.001
Department	0.55	42.848	5	<.001
Marital Status	0.46	29.426	2	<.001
Nationality	0.42	24.063	1	<.001
Age	0.29	11.854	3	.008

### 4.5 Association between sleep quality and workplace demographics

**Table 7** reports Cramér’s V, together with the underlying chi-square test, for each variable’s bivariate association with PSQI category. Work schedule showed the strongest association with sleep quality (V = 0.78, χ²(1) = 85.343, p <.001), followed by experience (V = 0.72), shift selection (V = 0.66), education (V = 0.63), department (V = 0.55), marital status (V = 0.46), nationality (V = 0.42), and age (V = 0.29, p = .008). These bivariate associations mirror the strong pairwise relationships among the exposure and covariate set (e.g., work schedule and shift selection co-occur strongly), which are formally quantified as collinearity in **Table 7**.

### 4.6 Collinearity diagnostics

**Table 8** reports variance inflation factors (VIF) for each dummy-coded predictor considered for the multivariable model (Section 3.4). Experience showed severe collinearity (VIF ≈ 13–14) and Age showed moderate collinearity (VIF ≈ 4–5), both driven by their strong association with Work Schedule and Shift Selection. Marital Status, Education, and Nationality showed acceptable VIFs (< 4). An exploratory model containing all covariates simultaneously with Work Schedule and Shift Selection failed to converge to stable estimates (Wald standard errors > 15, odds ratios exceeding 10^6^), consistent with quasi-complete separation arising from near-deterministic subgroups in the data (e.g., 38 of 42 nurses with ‘Other’ education, and 42 of 45 nurses with ≥10 years’ experience, reported good sleep). Education and Experience were therefore excluded from the primary adjusted model (**Table 6**) and are reported descriptively only (**Table 2**).

**Table 8:** Collinearity diagnostics (variance inflation factors).

Predictor	VIF
Age (25–29)	4.78
Age (30–34)	4.32
Age (>35)	5.05
Marital Status (Single)	1.75
Marital Status (Other)	1.13
Education (Diploma)	2.06
Education (Other)	3.91
Experience (1–5yr)	13.74
Experience (5–10yr)	13.13
Experience (≥10yr)	13.70
Nationality (Non-Saudi)	2.61
Work Schedule (Rotation)	3.52
Shift Selection (Mandatory)	3.53

### 4.7 Adjusted regression analysis of sleep quality replaces the PLS-SEM mediation model

**Table 9** presents the results of the pre-specified, parsimonious multivariable logistic regression for poor sleep quality (PSQI Poor vs. Good), adjusted for nationality and marital status and estimated using Firth’s penalized logistic regression to accommodate the sparse cross-tabulations documented above. The model showed good discrimination (McFadden’s pseudo-R² = 0.59). After adjustment, nurses working rotating shifts had substantially lower odds of poor sleep quality than nurses working fixed daytime schedules (OR = 0.02, 95% CI [0.00, 0.10], p <.001), and nurses who were mandatorily assigned to their shift had lower odds of poor sleep quality than those who selected their shift voluntarily (OR = 0.14, 95% CI [0.03, 0.61], p = .009). Neither nationality (OR = 0.28, 95% CI [0.05, 1.63], p = .156) nor marital status (Single vs. Married: OR = 1.82, 95% CI [0.50, 6.60], p = .362; Other vs. Married: OR = 0.45, 95% CI [0.02, 8.72], p = .599) was independently associated with sleep quality after adjustment.

**Table 9:** Adjusted logistic regression for poor sleep quality (Model A, primary).

Predictor	OR	95% CI	p
Shift Rotation (vs. Daytime)	0.02	0.00–0.10	<.001
Mandatory (vs. Voluntary)	0.14	0.03–0.61	.009
Non-Saudi (vs. Saudi)	0.28	0.05–1.63	.156
Single (vs. Married)	1.82	0.50–6.60	.362
Other marital status (vs. Married)	0.45	0.02–8.72	.599

Outcome: Poor Sleep Quality (PSQI) vs. Good. McFadden’s pseudo-R² = 0.59. Estimated by Firth’s penalized logistic regression (49, 50).

Two sensitivity models, individually adding Education (Model B: Supplementary Table-1) or Experience (Model C: Supplementary Table-2) to the primary model (Model A), left the direction and significance of the Work Schedule and Shift Selection estimates unchanged, though the added education/experience coefficients themselves were unstable (wide confidence intervals) and are not interpreted as independent effects, consistent with the collinearity documented in **Table 7**. Full sensitivity-model output is available in the accompanying analysis script (**Supplementary Materials**).

## 5 Discussion

This study examined cross-sectional associations between work schedule, shift selection, sociodemographic factors, and sleep quality among nurses in Saudi Arabia. The results show that sleep quality declines with age rather than improving, consistent with the positive age–PSQI association and with the adjusted model. This direction contrasts with Chung et al. (29), who reported that older age and longer nursing tenure were associated with a reduced likelihood of poor sleep; possible explanations for the discrepancy include differences in national and organizational context, sample composition, or accumulated occupational strain among older nurses in this cohort, and this warrants targeted investigation in future work.

Bivariate associations (**Table 7**) confirmed that work schedule, experience, shift selection, education, department, marital status, and nationality were all significantly associated with sleep quality, with Cramér’s V values indicating moderate-to-strong effects (0.29–0.78). Participants with ‘Other’ educational qualifications and ≥10 years of experience reported the highest rates of good sleep quality a pattern that runs counter to some prior work linking lower educational attainment to poorer sleep (30–32) and should be interpreted cautiously, since **Table 8** shows these variables are severely collinear with shift assignment (VIF up to 14) and cannot be disentangled from it in this sample.

The central finding of the adjusted model (**Table 9**) is that, after accounting for nationality and marital status, nurses on rotating shifts and nurses mandatorily assigned to their shift had substantially lower odds of poor sleep quality than nurses on daytime schedules or those who selected their shift voluntarily. This runs counter to the general direction reported in much of the shift-work literature, where rotating and non-standard schedules are typically associated with worse, not better, sleep outcomes (33–35). We did not remove or reverse this finding; it is a genuine, adjustment-robust pattern in these data and is discussed rather than explained away. Several non-mutually-exclusive explanations are plausible. First, a healthy-worker or selection effect may operate in a cross-sectional sample: nurses who tolerate rotating schedules poorly may transfer into daytime roles over time, leaving rotating-shift cohorts disproportionately composed of nurses who adapt well, while daytime ‘voluntary’ scheduling may include nurses managing other stressors (e.g., family or health circumstances) that independently impair sleep. Second, institutional scheduling practices in this setting may allocate structured rotation with built-in recovery time- preferentially to more experienced or resilient staff, while less structured ‘voluntary’ daytime arrangements may not carry the same protections; this would be consistent with the strong entanglement between shift assignment and experience documented in **Tables 3**-**4** and **7**. Third, residual confounding by education and experience, which could not be entered into the adjusted model due to collinearity, may still account for part of this association. Because the design is cross-sectional and adjustment was limited to two covariates, this finding should be read as an adjusted statistical association meriting prospective replication, not as evidence that rotating or mandatory scheduling protects sleep.

The Two-Process Model of Sleep Regulation (24, 25) is retained here strictly as background motivation for the Work Schedule association: night and rotating shifts can, in principle, misalign the circadian rhythm (Process C) and reduce homeostatic recovery (Process S). This study did not measure either process directly, and the model is not extended to interpret the education, experience, nationality, marital status, or department associations, which are sociodemographic and organizational rather than biological in nature.

Nationality and marital status, both significantly associated with sleep quality bivariately (**Tables 5**, **6**), were not independently associated with sleep quality once adjusted for shift exposure (**Table 6**). This pattern is consistent with nationality’s strong entanglement with shift assignment documented in **Table 3**, **4** (Saudi nurses were more likely to receive mandatory, rotating assignments) rather than an independent sociodemographic effect on sleep. This does not contradict prior work reporting ethnic or national differences in sleep (36–38); rather, it illustrates that in this sample such differences appear to operate largely through their association with shift assignment rather than independently of it.

Despite the counterintuitive direction of the rotating-shift finding, poor sleep quality remained common overall (57.1% of the sample), and healthcare institutions should continue to support nurses across all shift types in developing structured sleep strategies. Research indicates that nurses with good sleep outcomes maintain consistent sleep schedules during night shift periods by adjusting their sleep environment and following structured routines (39). Institutional support for sleep health should not be limited to nurses on rotating schedules based on this study’s adjusted findings alone; prospective, multi-site research is needed before these associations inform scheduling policy.

## 6 Strengths and limitations

This study’s principal strength lies in the size and specificity of its sample, comprising 140 nurses across multiple hospitals within a defined region. The research employed a validated instrument, the Pittsburgh Sleep Quality Index (PSQI), with an alpha coefficient of.824 in this sample, and utilized a fully reproducible, reviewer-audited analysis pipeline. Nonetheless, several limitations should be considered when interpreting these findings.

The cross-sectional design only permits associative, rather than causal, inferences. The temporal ordering assumed in the originally submitted mediation model was unsupported, as shift assignment, education, and experience were all measured at a single time point. The sample was predominantly female (96.4%), which limits the generalizability of the results to male nurses, who accounted for only 5 of 140 participants. Sleep quality was measured using the self-reported PSQI rather than objective measures such as actigraphy or polysomnography, which may introduce recall or reporting bias; however, the internal consistency of the instrument in this sample was satisfactory (α = .824). Education, experience, and age exhibited strong collinearity with the shift-exposure variables (see **Table 7**), precluding their simultaneous estimation in a single adjusted model. Consequently, their associations with sleep quality are presented descriptively (see **Table 2**) and should not be interpreted as independent, adjusted effects. The dataset lacked a site identifier, preventing the modeling of hospital-level clustering. Finally, although the attrition rate was notable (87 of 227 nurses, 38.3%), which raises concerns about potential non-random loss, this issue was addressed through transparent reporting—including a comprehensive CONSORT flow diagram—to enable a clear assessment of potential systematic differences and to uphold the validity of the study’s conclusions.

## 7 Conclusion

In this cross-sectional sample of 140 nurses in Saudi Arabia, work schedule and shift selection showed the strongest associations with sleep quality, and both remained significantly associated with lower odds of poor sleep after adjustment for nationality and marital status in a direction that runs counter to much of the shift-work literature and warrants cautious interpretation and prospective replication rather than immediate policy application. Education, experience, and age were strongly associated with sleep quality bivariately but could not be disentangled from shift assignment in an adjusted model due to collinearity. These findings underscore the importance of considering work-related and sociodemographic factors jointly when studying nurses’ sleep quality, and of using designs and analytic strategies suited to the temporal and structural constraints of the data at hand. Future research should employ prospective or longitudinal designs capable of establishing temporal order, collect hospital-site identifiers to account for clustering, and examine additional influences such as stress, physical activity, and mental health, in order to develop evidence-based strategies for improving sleep quality among shift-working nurses.

## Data Availability

The raw data supporting the conclusions of this article will be made available by the authors, without undue reservation.
