# Supplementary material for: Shift work, sociodemographic factors, and sleep quality among nurses working in rotating and day shifts: a cross-sectional analysis
Source: Front Psychiatry. 2026 Sep 3;17:1860489. doi: 10.3389/fpsyt.2026.1860489 (PMC13582511; doi:10.3389/fpsyt.2026.1860489)
Supplement: Supplementary file 1 [file Table1.docx]

**Supplementary materials**

**Model B**

| **Model** | **Predictor** | **coef** | **SE** | **OR** | **CI_lower** | **CI_upper** | **p** | **McFadden_R2** |
| --- | --- | --- | --- | --- | --- | --- | --- | --- |
| Model B (+education) | Intercept | 1.1354 | 0.8766 | 3.1124 | 0.5584 | 17.3479 | 0.19522 | 0.674 |
| Model B (+education) | WorkSchedule_Rotation | -5.441 | 1.4805 | 0.0043 | 0.0002 | 0.0789 | 0.00024 | 0.674 |
| Model B (+education) | ShiftSelection_Mandatory | -0.9653 | 1.4533 | 0.3809 | 0.0221 | 6.5747 | 0.50657 | 0.674 |
| Model B (+education) | Nationality_NonSaudi | 0.4983 | 0.8837 | 1.6459 | 0.2912 | 9.3034 | 0.57285 | 0.674 |
| Model B (+education) | Marital_Single | 0.8629 | 0.7068 | 2.3701 | 0.5931 | 9.4716 | 0.22212 | 0.674 |
| Model B (+education) | Marital_Other | -1.6612 | 1.576 | 0.1899 | 0.0086 | 4.1691 | 0.29184 | 0.674 |
| Model B (+education) | Edu_Diploma | 4.6774 | 1.7155 | 107.494 | 3.7252 | 3101.8593 | 0.0064 | 0.674 |
| Model B (+education) | Edu_Other | 0.9812 | 1.8374 | 2.6678 | 0.0728 | 97.7564 | 0.59331 | 0.674 |

**Model C**

| **Model** | **Predictor** | **coef** | **SE** | **OR** | **CI_lower** | **CI_upper** | **p** | **McFadden_R2** |
| --- | --- | --- | --- | --- | --- | --- | --- | --- |
| Model C (+experience) | Intercept | 0.9788 | 1.9588 | 2.6614 | 0.0573 | 123.7172 | 0.61727 | 0.623 |
| Model C (+experience) | WorkSchedule_Rotation | -2.9895 | 0.8494 | 0.0503 | 0.0095 | 0.2659 | 0.00043 | 0.623 |
| Model C (+experience) | ShiftSelection_Mandatory | -1.3005 | 0.7753 | 0.2724 | 0.0596 | 1.2448 | 0.09344 | 0.623 |
| Model C (+experience) | Nationality_NonSaudi | -0.6438 | 0.8704 | 0.5253 | 0.0954 | 2.8924 | 0.45948 | 0.623 |
| Model C (+experience) | Marital_Single | 0.5951 | 0.7253 | 1.8133 | 0.4376 | 7.5135 | 0.41192 | 0.623 |
| Model C (+experience) | Marital_Other | -1.0447 | 1.4236 | 0.3518 | 0.0216 | 5.7293 | 0.46305 | 0.623 |
| Model C (+experience) | Exp_1_5 | 1.4286 | 1.8511 | 4.1728 | 0.1109 | 157.0732 | 0.44026 | 0.623 |
| Model C (+experience) | Exp_5_10 | 1.8287 | 1.8814 | 6.226 | 0.1558 | 248.7246 | 0.33106 | 0.623 |
| Model C (+experience) | Exp_ge10 | -0.3334 | 2.0015 | 0.7165 | 0.0142 | 36.2185 | 0.86771 | 0.623 |
